# Bioprospecting for polyesterase activity relevant for PET degradation in marine Enterobacterales isolates

**DOI:** 10.3934/microbiol.2023027

**Published:** 2023-06-15

**Authors:** Denisse Galarza–Verkovitch, Onur Turak, Jutta Wiese, Tanja Rahn, Ute Hentschel, Erik Borchert

**Affiliations:** 1 GEOMAR Helmholtz Centre for Ocean Research Kiel, Kiel, Schleswig-Holstein, Germany; 2 Christian-Albrechts University of Kiel, Kiel, Schleswig-Holstein, Germany

**Keywords:** marine bacteria, order Enterobacterales, PETase-like activity, sponges, bryozoans

## Abstract

Plastics have quickly become an integral part of modern life. Due to excessive production and improper waste disposal, they are recognized as contaminants present in practically all habitat types. Although there are several polymers, polyethylene terephthalate (PET) is of particular concern due to its abundance in the environment. There is a need for a solution that is both cost-effective and ecologically friendly to address this pollutant. The use of microbial depolymerizing enzymes could offer a biological avenue for plastic degradation, though the full potential of these enzymes is yet to be uncovered. The purpose of this study was to use (1) plate-based screening methods to investigate the plastic degradation potential of marine bacteria from the order Enterobacterales collected from various organismal and environmental sources, and (2) perform genome-based analysis to identify polyesterases potentially related to PET degradation. 126 bacterial isolates were obtained from the strain collection of RD3, Research Unit Marine Symbioses-GEOMAR-and sequentially tested for esterase and polyesterase activity, in combination here referred to as PETase–like activity. The results show that members of the microbial families *Alteromonadaceae*, *Shewanellaceae*, and *Vibrionaceae*, derived from marine sponges and bryozoans, are the most promising candidates within the order Enterobacterales. Furthermore, 389 putative hydrolases from the α/β superfamily were identified in 23 analyzed genomes, of which 22 were sequenced for this study. Several candidates showed similarities with known PETases, indicating underlying enzymatic potential within the order Enterobacterales for PET degradation.

## 1. Introduction

Marine microbial communities play a key role in the maintenance and support of the marine ecosystems health [1]. Due to the diversity of interactions, including their adaptation to extreme conditions [2] and host–associated interactions [3], marine microorganisms have evolved a remarkable variety of enzymatic systems that are involved in many biological reactions [4], and exhibit novel biocatalytic properties [4],[5]. By leveraging their ability to catalyze biological reactions, marine microorganisms have the potential to be used as a cost–effective and sustainable way to reduce pollution and mitigate the negative impacts caused by mankind.

Plastics have become an important part of our daily lives. Their versatility, durability, and convenience (i.e., low–cost production and disposable nature) have led us to become increasingly dependent on them. It is estimated that plastic production reached 390 million metric tons (Mt) worldwide in 2021 [6]. Due to disposal mismanagement, about 4 to 12.7 Mt of plastics ultimately end up in the ocean every year [7]. Here, they are integrated into various trophic levels, predominantly in the form of micro– and nanoparticles (size < 5 mm) [8],[9], and associated with the spread of contaminants and invasive species [10]–[14].

Polyethylene terephthalate (PET) is a widely produced polymer used in a variety of applications [15],[16]. By 2021, the annual production exceeded 30 Mt [6]. Recycling strategies such as mechanical or chemical degradation have been proposed [17]–[19], but these methods often involve high energetic costs or/and are limited to specific operative requirements. In contrast, biodegradation strategies offer a more energy–efficient and environmentally friendly recycling alternative, and for PET one of the best–characterized depolymerization enzymes described to date is available [20]–[24]. PET–active enzymes belong to carboxylic ester hydrolases (EC 3.1.1), which also include cutinases (EC 3.1.1.74), lipases (EC 3.1.1.3), and esterases (EC 3.1.1.1) [25]. Most of the known PET–active enzymes have a bacterial origin [26], these hydrolases have been described from the terrestrial thermophilic *Thermobifida fusca* [27] and the mesophilic *Ideonella sakaensis* [28], to name the most prominent examples. Only a few have been found in eukaryotes. One such example is the fungus *Aspergillus*, with several species from different habitats being reported to degrade various types of plastic [29]. PET–acting enzymes are generally characterized as serine hydrolases that typically have a catalytic triad in their active site (Ser-His-Asp) and an α/β–hydrolase fold [1],[30]. Functionally verified PET hydrolases have shown to contain a canonical C-terminal disulfide bond, whereby some having an additional disulfide bond elsewhere as well [30]–[32].

In recent years, marine–associated microorganisms have gained attention due to their ability to degrade PET [26]. Nevertheless, their degradative properties remain to be identified for many taxa, and the mechanisms involved are still unknown [33]. However, by utilizing existing strain collections it is possible to investigate the degradation potential of different microbial groups. Gambarini and colleagues found that a significant proportion (15.9%) of the plastic–degrading organisms described in the literature were derived from strain collections, with bacterial groups from the Proteobacteria phylum (30.4%) showing promise [33].

The order Enterobacterales consists of a large and diverse group of bacteria defined as non–spore–forming, gram–negative, facultative anaerobic, and belonging to the class Gammaproteobacteria [34]. Members of this group inhabit a variety of ecological niches and are found in both terrestrial and aquatic environments –freshwater and marine, and in association with a wide range of living organisms [35],[36]. There are 15 defined bacterial families in the class Enterobacterales, of which characteristic genera such as *Alteromonas* [37], *Colwellia* [38], *Enterobacter* [39]–[41], *Pseudoalteromonas* [42]–[44], *Shewanella* [45], *Thalassomonas* [44],[46], and *Vibrio* [44] have been described as capable of degrading complex natural hydrocarbons such as chitin, collagen, agar, and cellulose. Furthermore, they belong to the unique group of so–called obligate hydrocarbonoclastic bacteria (OHCB), which are responsible for most of the natural degradation of hydrocarbon contaminants in the marine ecosystem [47]. Previous reports indicate that members of the above–mentioned families can display cutinase, esterase, and lipase activity. These enzymes are closely related to the degradation of PET and represent an interesting group that should be investigated for their degradation potential [39],[41],[42],[45],[47].

The main objective of this study was to investigate selected marine Enterobacterales strains from our strain collection to reveal potential enzymatic activities of biotechnological relevance. A standardized agar plate–based screening was employed to evaluate microbial PETase-like activity [48],[49]. Bioinformatical tools were then used to generate a phylogenetic tree and profile the detected PET hydrolase related activity. The study revealed that the main candidates for PETase–like activity are bacterial strains belonging to the genera *Pseudoalteromonas, Photobacterium*, and *Vibrio* associated with marine sponges and bryozoans.

## 2. Materials and methods

### 2.1. Bacterial selection and cultivation

#### 2.1.1. Selection

In this study, a total of 125 bacterial strains of the order Enterobacterales were selected from the strain collection of our Research Unit Marine Symbiosis to evaluate their potential for degrading PET. The selection process involved combining a neighbor–joining reference tree of potential PET hydrolase activities generated by Danso and colleagues in 2018 [26] with a targeted search of the genome–based Taxonomy Database for Prokaryotic Genomes (GTDB) [50]. This approach enabled the identification of putative strain candidates with the highest potential for PET degradation based on their evolutionary relationships and taxonomic classification.

Additional parameters, such as the marine source the strain was isolated from, were recorded. A variety of habitats and associated organism sources–algae, bryozoans, cnidarians, hydrozoans, bivalves, nematodes, sediment, sponges, fishes, and seawater–were included (Supplementary Material Table S1). In addition, bar graphs were created using RStudio (version 2022.7.1.554) [51] to show the source and activity distributions of the strains tested relative to their family–level identity.

#### 2.1.2. Taxonomic confirmation and phylogenetic tree construction

Pure bacterial strains were cultured on agar plates with marine broth (MB) (BD Difco™ 2216; 37.4 g/L) (Bacto™ agar; 15 g/L) and incubated for one week at room temperature. In parallel, colonies were taken from each isolate as a subsample to check taxonomic status. DNA was extracted using the DNeasy® Blood & Tissue Kit (QIAGEN®, Germany) according to the manufacturer's instructions. Primers 27F (5′-GAGTTGATCCTGGCTCAG-3′) and 1492R (5′-GGTTACCTTGTTACGACTT-3′) were used to amplify 16S rRNA gene fragments [52],[53]. Gene fragments were verified by agarose gel electrophoresis (1% w/v peqGOLD universal agarose in 1 x TAE buffer) and sequenced by Eurofins (Eurofins Umwelt Nord GmbH Branch, Kiel, Germany).

For phylogenetic tree construction, the 16S rRNA gene sequences were merged into a FASTA–formatted file using MEGA-X [54]. The sequences were aligned using the Neighbor–Joining method (NJ) with ClustalW2 [55]. The phylogenetic tree was constructed using FastTree [56],[57]. The Interactive Tree of Life [58] was used to visualize the tree, and the Annotation Editor Tool (iTOL version 6.5.8) [59] was used to record the corresponding metadata.

### 2.2. Functional screening using polymer-based indicator plates

**Figure 1.**
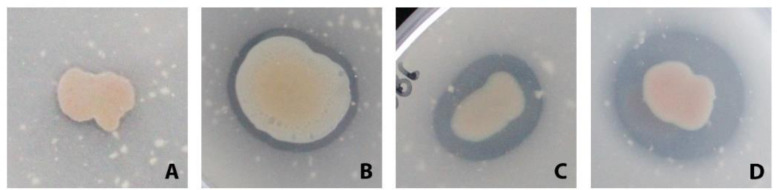
Graphical reference for activity classification on agar-plate substrate media. Clearance area category example after 10 days on tributyrin agar plates. (A) no activity, (B) low–activity, (C) medium–activity, and (D) high–activity. This reference area was used as an approximate to classify activity for all media tested.

A modified version of the standardized agar plate method to detect polyesterase activity of Molitor et al. (2020) was used to screen the selected strains. An initial screening using tributyrin (TRI, 1.5% v/v in MB) as a substrate was performed to establish the presence of general esterase activity. To determine more specific polyesterase and “PETase–like” activity, two additional aliphatic polyester substrates, polycaprolactone diol (PCD Mn530, 1.5% v/v in MB) and polycaprolactone (PCL, 1,5% v/v in MB) were tested [48]. Additional testing was performed to confirm the presence of PET–like activity with bis(2-hydroxyethyl) terephthalate (BHET, ~0.5 mM v/v in MB) using the methodology described by Perez-Garcia and colleagues [49]. Bacterial isolates were tested in triplicate. Positive isolates showed a clear halo after 10 days of incubation at room temperature, and additional activity controls were performed after three, five, and seven days of incubation (Figure 1). Based on the results, the activity was categorized as follows: low activity (+), medium activity (++) and high activity (+++). Furthermore, in order to discriminate between PETase-like and PETase activity, PET nanoparticle media was produced (PETnp, ~0.3 mg/mL in MB) using the methodology described by Perez-Garcia and colleagues [49]. Finally, PET foils (Goodfellow, ES301445/8) were incubated with 8 ml of MB medium with the corresponding isolate at room temperature (21 °C) for 30 days. After incubation, the foils were washed with water, followed by two washes with 100% ethanol. They were then dried at 37 °C for 48 hours and the weight was recorded subsequently.

### 2.3. Mining for PETase-like enzymes

#### 2.3.1. Oxford Nanopore Technologies™ whole–genome–sequencing

After plate–based screening, 23 candidates with prominent polyesterase activity were confirmed and selected. 22 were chosen for genome sequencing, and one additional was obtained from NCBI database (DSM 3368^T^) (Accession number: GCA_001083805.1). High molecular weight DNA was extracted using a sample preparation and lysis protocol described in the Qiagen® Genomic DNA Handbook and purified using the Genomic-Tip 100/G (QIAGEN®, Germany) column. The quality of the DNA extraction was checked by agarose gel electrophoresis (2% w/v peqGOLD universal agarose in 1 x TAE buffer) and quality was assessed using the A260/280 ratio of a NanoDrop™ spectrophotometer [60]. All 22 strains of interest were sequenced using the Rapid Sequencing Kit® (RAD-004) according to the manufacturer's specifications. MinION Flongle flow cell loaded with the extracted genomic DNA was set at 230 Hz, 35 °C for 24 hours. The respective base calling was performed by converting the raw sequence data (FAST5) into FASTQ files, which was done using Guppy software (version 2.3.1 from Oxford Nanopore Technologies™).

### 2.4. Bioinformatic analyses

#### 2.4.1. Nucleic acid quality control and taxonomy

FASTQ sequence files obtained with the Guppy software were used to generate a genome assembly using Flye software (version 2.8.2) [61]. The resulting genomes were first used to assess the quality of the sequenced genomes using CheckM [62]. To accurately classify the genomes, the Genome Taxonomy Database Toolkit (GTDB-Tk v2.0.7) [63] was employed. In GTDB-Tk, genes were called using Prodigal [64] and bacterial marker genes were identified using HMMER [65]. Sequences were then inserted into a domain–specific reference tree using pplacer [66]. Taxonomic classification was based on a combination of sequence placement within the GTDB, its relative evolutionary divergence (RED), and average nucleotide identity (ANI) with the reference genomes [62].

#### 2.4.2. Protein analysis and homology modeling

FASTA nucleotide files were translated with Prodigal (v2.6.3). Functional annotation of the predicted protein output was performed with EggNOG mapper (v 2.0) using eggNOG database v.5.0 [67]. A database of potential PETase proteins was created from all 22–genomes sequenced plus one genome from NCBI database (DSM 3368^T^/ATCC 29999, accession number FMWJ00000000). The query criterion included all ortholog group hits belonging to domains of the superfamily of α/β–hydrolases_5 (pfam12695). Following the method for genomic enzyme mining proposed by Almeida and colleagues. (2019) [68], phylogenetic analyses were conducted on the selected protein sequences with MEGA-X [54] using maximum likelihood statistical method and WAG + G model, with 1000 bootstrap replications with 50% bootstrap cut–off value. A reference dataset was conducted in parallel with the phylogenetic analysis, which integrated from the plastics–active enzymes database–PAZy [69] including 10 PET hydrolases and homolog polyesterases from the Gammaproteobacteria, and *Is*PETase (ISF6_4831) from *Ideonella sakaensis* [28]. Both were used to create a custom BLASTP dataset.

#### 2.4.3. Genome mining

The amino acid sequences of interest were aligned using T-COFFEE Expresso [70]. Final outputs were represented and analyzed using ESPript 3.0 [71], and the Lipase Engineering Database–LED [72] served to identify the catalytic triad and oxyanion residues. SignalP 5.0 [73], was used to estimate cleavage sites for secretory protein signal peptides to indicate the specific localization of the enzymatic activity. Finally, structural homologs were confirmed using 3D structure comparisons with RoseTTAFold software [74].

## 3. Results

### 3.1. Overview of bacterial isolates

**Figure 2.**
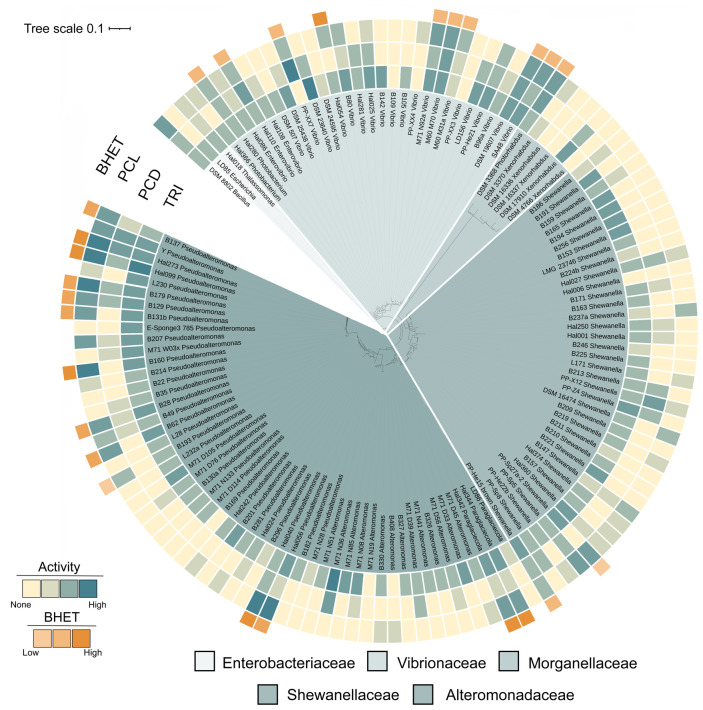
Phylogenetic tree and enzymatic activity screening results for all isolates screened (n = 126). Phylogenetic relatedness of the 5 tested families of the order Enterobacterales is displayed. The screening results are displayed from the inside out for the respective activities on TRI, PCD, PCL and BHET. As outgroup we used DSM 8802^T^ *Bacillus halotolerans*.

Bacterial isolates used in this study covered five families within the order Enterobacterales: *Alteromonadaceae* (53 isolates), *Enterobacteraceae* (2 isolates), *Morganellaceae* (6 isolates), *Shewanellaceae* (37 isolates), and *Vibrionaceae* (27 isolates). The family *Alteromonadaceae* accounted for the greatest number of isolates, including representatives from the genera *Alteromonas* (15), *Paraglaciecola* (3), and *Pseudoalteromonas* (35). The family *Shewanellaceae* had the second-most abundant number of isolates, with *Shewanella* (37). As for *Vibrionaceae*, more restrictive selection criteria were employed to comply with laboratory safety rules, and only strains belonging to the risk group 1 were used. The two genera tested were *Photobacterium* (2) and *Vibrio* (25). *Morganellaceae* isolates included representatives of *Xenorhabdus* (6). The family *Enterobacteraceae* covering *Escherichia* (1), and *Thalassomonas* (1) (Figure 2). Each isolate was inoculated onto all four MB agar substrates. Additional strain information, such as isolation source is shown in Supplementary Material Table S1.

### 3.2. Functional screening using polymer–based indicator plates

Based on the size of the clearing halo area, all strains tested were classified in categories: “–, no activity”, “+, low activity,” “++, medium activity” or “+++, high activity”. For the esterase enzyme screening with tributyrin MB–enriched media, 71 had low activity, 41 had medium activity, and 3 demonstrated high activity. The high activity strains were M71_N36 (*Alteromonas* sp.), Hal099 (*Pseudoalteromonas* sp.), and DSM 23640^T^ (*Vibrio caribeanicus*). A total of 11 isolates were not active on tributyrin–MB plates, although all of them grew on the media. For polyesterase activity on PCD supplemented MB agar plates, 69 isolates had no activity, 48 presented low activity, 8 had medium activity, and one (strain PP-XX7, *Vibrio* sp.) revealed high activity. PCL–supplemented MB agar plates were used to test for PETase–like activity. 69 isolates had no activity, 35 presented low activity, 17 had medium activity, and 6 (all representatives from the family *Alteromonadaceae*, strains: Hal273, Hal099, Hal040, Hal056, B160, and B179) revealed high activity.

For the esterase assay on BHET–supplemented MB agar plates, a total of 59 strains, which presented some activity for PCL, were tested for activity on BHET MB–enriched media. 37 isolates were inactive, 11 presented low activity, 6 had medium activity, and 8 revealed high activity. 24 of the BHET active isolates were further used for PETnp plate incubation test, of which five displayed high activity, two medium activity, four slight activity and 13 no activity (Supplementary Material Table S2). 11 of the 24 BHET active strains were further used for a liquid incubation assay of the strains in MB media with PET foil. After 30 days of incubation at room temperature (21 °C) no weight loss could be determined, but rather a slight weight increase in the range of 0.5–3% (Supplementary Material Table S3).

### 3.3. Isolates source–based classification

**Figure 3.**
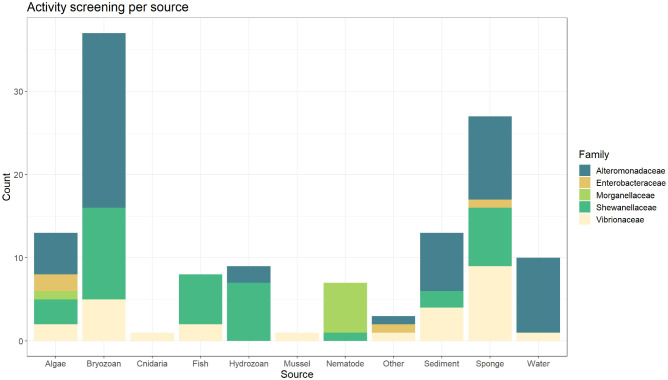
Distribution of isolates obtained from different sources and their respective taxonomic affiliation within the order Enterobacterales. The stacked bar plot shows the number of isolates tested by their origin sources, where isolates obtained from bryozoans and sponges build the majority. The plot also shows the counting results in relation to bacterial family representatives per source.

The source of each strain was documented for each of the isolates. Almost all strains tested were associated with a different host organism, though there were samples obtained from the water column, sediment, and one strain isolated from biofilm on marine bone experiments (strain SA48) in the Kieler Fjord. There were 11 source categories (Figure 3) covered: algae (11), bryozoans (36), cnidaria (1), fish (8), hydrozoans (9), mussels (1), nematodes (7), sediment (13), sponges (27), and water (10). There were three strains from biofilm collections, one from marine bone degradation experiments (strain SA48) and the other two purchased from the DSMZ-German Strain Collection of Microorganisms and Cell Cultures, (strains DSM 498^T^, and DSM 507^T^). According to the database these strains were included in the category “other”. As shown in Figure 3, strains isolated from bryozoans (36) and sponges (27) dominate in the number of strains tested per source. Both sources present strains with polyesterase related activity. Strains originating from bryozoans are widely distributed throughout the tree; represented families include *Alteromonadaceae* (17), *Shewanellaceae* (11), and *Vibrionaceae* (5). Sponge isolates were the most diverse, with members from *Alteromonadaceae* (10), *Vibrionaceae* (7), *Shewanellaceae* (7), and *Enterobacteraceae* (1).

### 3.4. Mining for PETase–like activity

#### 3.4.1. Taxonomic identification and DNA quality control

Following positive results on BHET MB–enriched media, 23 isolates were selected for their potential polyesterase activity for genome sequencing, as well as from NCBI database (DSM 3368^T^). Genomic DNA was extracted and MinION™ device–based sequencing (Oxford Nanopore Technologies) was performed for the 22 isolates selected, utilizing one Flonge flow cell (Oxford Nanopore Technologies) per genome. The correct taxonomic identification was obtained using GTDB-Tk (Table 1) for all 23 final genomes analyzed.

**Table 1.** GTDB-Tk taxonomic validation. Results confirming the taxonomical identity for the strains of interest. Strains with the same species level identity are marked with an asterisk (*). All listed strains were genome sequenced using the MinION device, besides DSM 3368^T^ as a high–quality genome was available online (Accession number: GCA_001083805.1). The enzymatic activities were categorized as follows: low activity (+), medium activity (++) and high activity (+++).

Strain ID	Closest–relative according to GTDB-Tk	TRI	PCD	PCL	BHET
B129*	*Pseudoalteromonas* sp.	++	+	++	+
B131b	*Pseudoalteromonas* sp.	++	-	++	++
B137	*Pseudoalteromonas* sp.	+	+	++	++
B160	*Pseudoalteromonas* sp.	++	-	+++	+++
B28	*Pseudoalteromonas* sp.	++	+	++	++
B62*	*Pseudoalteromonas* sp.	++	-	++	++
B193	*Pseudoalteromonas* sp.	+	-	+	++
DSM 3368^T^	*Photorhabdus luminescens*	+	++	++	+
DSM 19607^T^	*Vibrio coralliilyticus*	++	++	++	+
Hal110	*Enterovibrio sp*.	+	++	+	+
Hal040	*Pseudoalteromonas* sp.	+	+	+++	+++
Hal054	*Vibrio* sp.	+	-	+	+++
Hal056	*Pseudoalteromonas* sp.	++	+	+++	+++
Hal099	*Pseudoalteromonas* sp.	+++	+	+++	+++
Hal273*	*Pseudoalteromonas* sp.	++	++	+++	+++
Hal280	*Photobacterium* sp.	+	+	+	+
Hal342	*Paraglaciecola* sp.	++	+	++	+++
M60_M31a	*Vibrio* sp.	+	+	+	+
M60_M70	*Vibrio* sp.	++	++	++	+
PP-He15brown	*Shewanella* sp.	++	+	+	+
PP-Sp27a-2	*Shewanella* sp.	++	-	++	+
PP-XX7	*Vibrio* sp.	-	+++	+	+
SA48	*Vibrio* sp.	++	+	++	+

Five genera were linked to potential PETase–like activity in this study including: *Vibrio* (8), *Pseudoalteromonas* (12), *Shewanella* (2), as well as one representative strain for *Photorhabdus* and *Photobacterium*. The respective genome quality, purity, size, and predicted genes for the aforementioned selected strains are displayed in detail in Supplementary Material (Table S4).

### 3.5. Computational protein analyses

Sequences related to esterase activity were extracted from the 23 genomes, yielding 389 sequences of interest. These sequences and known PETases (Supplementary Material Table S5) were included for comparison in a phylogenetic tree (Supplementary Material Figure S6).

Results from the phylogenetic survey highlighted three groups of interest, each group associated with a previously identified PETase enzyme (Table 2). Group one included two predicted esterases from strains Hal110 and Hal280 related to ISF6-4831, isolated from *Ideonella sakaensis* [28]. The second group is a complex of hydrolase proteins from isolates B160, Hal099, and Hal110, which were associated with PmC cutinase isolated from *Pseudomonas mendocina* [77]. Group three was associated with Poly(3-hydroxybutyrate) depolymerase (EC 3.1.1.75), Poly(3-hydroxybutyrate) is a biodegradable alternative to plastics used in packaging and cosmetic industry [80]. The associated sequences originated from the strains Hal054, Hal280, and Hal342.

**Table 2.** PETase homologs from the sequenced isolates and their respective closely related reference sequences obtained from EggNOG mapper (v 2.0). Hydrolase sequences from six strains that were linked to confirmed PETase and PHB depolymerase activity.

	PETase or homolog activity	Strain ID	Query	Predicted function	e-value	Score
1	ISF6-4831 (*Ideonella sakaensis*)	Hal110	contig_69_62	Putative esterase	2.81e-229	635.0
		Hal280*	scaffold_3_1199	Carboxylesterase	3.91e-119	352.0
2	PmC (*Pseudomonas mendocina*)	B160	contig_2_38	Hydrolases or acyltransferases	1.6e-167	471.0
		Hal110	contig_69_78	Serine dehydratase	0.0	921.0
		Hal099*	contig_64_11	Hydrolase	4.05e-34	124.0
3	PHB depolymerase activity	Hal342	contig_2_32	Esterase PHB depolymerase	8.36e-107	315.0
		Hal280	contig_4_1213	Esterase PHB depolymerase	3.55e-161	462.0
		Hal342	contig_2_31	Esterase PHB depolymerase	5.89e-146	418.0
		Hal054	contig_1_1477	Putative esterase	1.85e-162	453.0

*PETase homologs selected for multiple sequence alignment.

**Figure 4.**
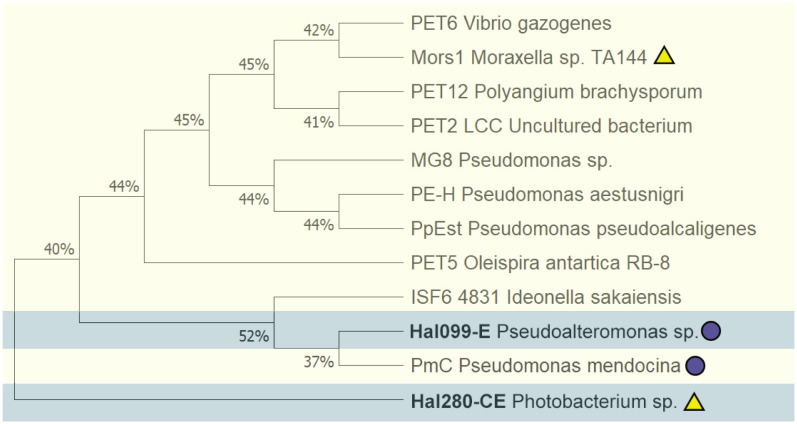
Maximum–likelihood (ML) phylogenetic tree for esterase sequences from Hal099-E and Hal280-CE using the reference dataset of PETase enzymes. The light–yellow background highlights all reference sequences listed in Table 2, and with light–blue the two protein sequences of interest are shown. The matched relatedness between the sequences is indicated by colour coded purple circles and yellow triangles, as determined by the LED database analyses.

The ortholog protein sequences of interest were screened using the LED database to determine their respective fold homolog. Through this process two protein sequences, Hal280-CE (a carboxylesterase from *Pseudoaltermonas atlantica*) and Hal099-E (an esterase from *Photobacterium sp*.), were identified as related to Mors1 lipase type II and PmC cutinase, respectively. To confirm their relatedness, all reference enzymes were grouped together, and their relationship is shown in Figure 4.

In a maximum–likelihood phylogenetic tree with reference PETases, both protein sequences of interest are placed outside of the clade. The signal peptide sequence found in Hal099-E suggests a potential for extracellular localization with a cleavage site between position 17 and 18, and was identified as Sec/SPII with >99% probability. Hal280-CE showed signal for a not specified type signal peptide (Supplementary Material Table S7).

**Figure 5.**
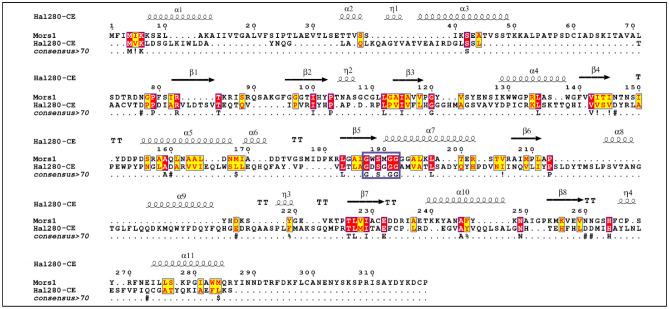
Amino acid sequence alignment of Hal280-CE polyesterase and Mors1. Hal280-CE was suggested as a suitable structural homolog for Mors1 by the LED server. Amino acid residues coloured in red correspond to the ones strictly conserved between Mors1 and Hal280-CE, while residues highlighted in yellow show areas with an average level of homology. The pentapeptide motif G-x1-S-x2-G is framed and highlighted by a purple rectangle.

Multiple sequence alignments (MSA) were generated using T-COFFEE Expresso and rendered with ESPript 3.0 to incorporate structural information including the location of the alpha helices and beta sheets linked to the protein sequences. Mors1 was selected for amino acid comparison with Hal280-CE (Figure 5). The resulting MSA shows that 30% of the amino acids in Hal280-CE were identical to Mors1, 2.8% were conserved. Hal280-CE contains the pentapeptide motif G-x1-S-x2-G, which is typical for previously reported polyesterases [1]. Instead of a Trp at position x1 and Met at position x2 in Mors1, Hal280-CE presents an Asp at x1 and a Gly at x2.

**Figure 6.**
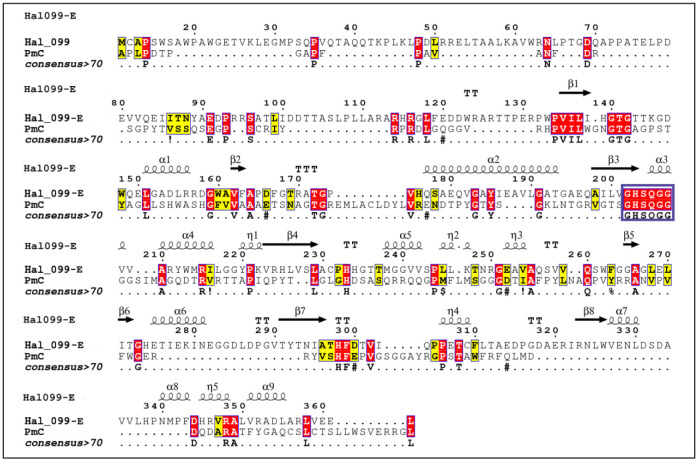
Amino acid sequence alignment of Hal099-E and PmC. Hal099 was suggested as a suitable structural homolog for PmC by the LED server. Amino acid residues coloured in red correspond to the ones strictly conserved between PmC and Hal099, while residues highlighted in yellow show areas with an average level of homology. The pentapeptide motif in G-x1-S-x2-G is fully preserved and shown and framed with the purple rectangle.

PmC was selected for amino acid comparison with Hal099-E (Figure 6). The resulting MSA inferred only 19% of the amino acids were identical to PmC, and 1.5% conserved. The pentapeptide motif in G-x1-S-x2-G is present. There is a beta sheet followed by an alpha helix where the pentapeptide is located, this is characteristic for polyesterase activity associated enzymes [1]. Structural homolog analysis was limited due to the short length of the sequence.

## 4. Discussion

In this study, the method described by Molitor and colleagues [48] and Pérez–García and colleagues [49] was adapted to investigate potential polyesterase activity associated with the degradation of PET. The strain selection criteria proved successful in finding potential candidates within the order Enterobacterales. Of the 126 strains tested, 91% showed positive esterase activity when tested for tributyrin. These positive results are associated with a variety of enzymatic activities, such as acyltransferases, lipases, polyesterases, and other hydrolases [48], not all of which are necessarily associated with PETase–like activity. To identify polyesterase activity potentially associated with degradation of PET, three substrates targeting this activity were investigated. PCD was used to detect polyesterase activity, since PCD is a liquid, emulsifiable, low molecular weight derivate from PCL. It provided a suitable assay to test polyesterase activity without the use of potentially toxic solvents [48]. However, the results of this plate–based assay were inconsistent, as 45% of the strains tested did not grow on the media, and of those that did grow, 22% were not active. PCL was used as a second substrate to detect polyesterase activity, with polycaprolactone (PCL) described as the standard substrate for this purpose [48],[68]. Because of its semicrystalline structure and the similarity of its physical and chemical properties to PET, this polyester was selected to demonstrate PETase–like activity [48].

The strains tested with high activity in all media tested (TRI, PCD, PCL, and BHET) were members of the genus *Pseudoalteromonas*, isolated mainly from sponges and bryozoans. Liu and colleagues (2019) previously described the PCL–degrading capacity of marine *Pseudoalteromonas* members, which was coincidentally associated with positive tributyrin activity in most cases [81]. It remains clear that the combination of tributyrin and PCL screening may provide a promising approach to detect esterase and polyesterase activity. Overall, 54% of the selected strains were inactive on PCL–enriched media. However, all strains tested with high PCL activity were also active towards BHET (bis[2-hydroxyethyl]terephthalate), which is an intermediate product in the generation and degradation of PET. BHET is one of the major degradation products of PET films and fibres [82]. The BHET activity screening was a successful step toward assessing PETase–like activities that belong to the category of PETase group I or PET surface–modifying enzymes [25]. PETases from this group have already attracted the attention of the industry, as they can be used to produce polyester textiles that are predominantly based on PET [25].

Furthermore, the identification of polyesterase activity under the given culture conditions (25 °C) could indicate a mesophilic nature of the PETase–like activity found, where optimal degradation activity for these enzymes could potentially occur at temperatures between 30 and 45 °C [26]. Whether this is the case depends on each bacterial strain, their optimal growth temperature, and their enzymatic properties. The results obtained in this study show that 20% of the strains tested were active on BHET substrate plates, of which 53% belonged to *Pseudoalteromonas*, 32% to *Vibrio*, 7% to *Shewanella*, as well as one representative of *Photobacterium* and *Photorhabdus*, suggesting a possible taxonomic relationship of polyesterase activity within the order Enterobacterales. The BHET positive strains were further tested on PET nanoparticle containing agar plates and for their capacity to reduce the weight of low-crystallinity PET foil in a liquid assay. While some isolates produced noticeable halos on PETnp plates (Supplementary Material Figure S8 and Table S2), none of the isolates lead to a weight reduction of PET foil over 30 days (Supplementary Material Table S3). The detected halo formation on PETnp plates might be attributable to residual agarose activity of the isolates, leading to a false-positive result. The results of these assays are therefore inconclusive and true PETase activity remains to be proven.

**Figure 7.**
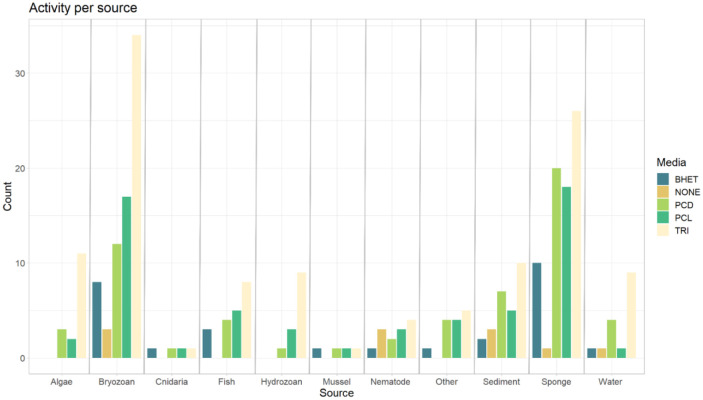
Substrate activity per tested source. The stacked bar plot shows the activity count accordingly to the tested media for lipase (TRI), polyesterase (PCD and PCL), carboxylesterase–like (BHET), and absence of activity where the count is depicted by origin source.

Interestingly, most of the positive screen results for polyesterase activity (Figure 7) were isolated from sponges (7) and bryozoans (6). Others also reported potential polyesterase activity in the microbiome of these organisms. In a recent study by Carr and colleagues (2022) [1], a cutinase–like polymerase (BgP) and further putative PETase genes were confirmed to be present in five isolates from deep–sea sponges [1]. The authors described holobiont–associated bacterial species as a possible source of PETase activity. Another example of sponges associated microorganisms with related PETase–like activity was studied by Almeida and colleagues [68]. The authors found a *Streptomyces* sp. strain (SM14) isolated from the sponge *Haliclona simulans* with synthetic polyester degradation activities. The presence of collagenolytic enzymes in sponges and their relationship with the recycling of spongins –collagen like fibrillar proteins– [83], might be involved in the degradation of other natural hydrocarbons [44] possibly also including polymer degradation.

In addition, bryozoans have been frequently associated with plastic litter in marine environments. These small aquatic invertebrates have been observed attached to various types of polymers [84],[85], which likely contributes to their widespread dispersal by plastic debris in the ocean [86]. It is therefore not surprising that symbiotic microorganisms from these hitchhikers of plastic debris might develop the ability to utilize plastic polymers as a carbon source, even just as a moon-lighting activity of other secreted enzymes. Recent research has revealed a potential link between bacteria isolated from marine symbiotic species and the presence of polyesterase activity [1],[68], which can be attributed to the diverse and metabolically varied communities that often coexist in microbial environments, rather than single, genetically identical individuals [87]. This functional diversity may enable ecological and evolutionary adaptations of microbial communities, conferring specific advantages such as increased energy efficiency and resistance to disturbances [88]. These findings could have important implications for the development of effective plastic debris mitigation strategies, including the optimization and testing of alternative recycling models [43],[79],[89]. The most prominent example of such an adaptation albeit perhaps being an exception, is the bacterium *Ideonella sakaiensis*, which was isolated from a sample stemming from the proximity of a plastic recycling plant [28].

After orthologue sequences were selected from 23 genomes, 389 protein sequences belonging to the α/β hydrolase superfamily, which includes proteins with esterase and lipase activity, were identified. When looking at the maximum likelihood phylogenetic tree (Supplementary Material, Figure S3) the potential of hydrolase activity related to polyesters is shown to be widely distributed in the order Enterobacterales. In fact, the proteins affiliated with the α/β hydrolase superfamily have been described to have several functions, acting as hydrolases, lyases, transferases, hormone precursors or transporters, and as chaperones for other proteins [90],[91]. The agar–plate screening methodology used in this study indicates that there is a considerable amount of polyesterase activity in the tested strains, suggesting that further research into true PET degradation might be worthwhile. The combination of experimental assays and computational tools helped to reveal the potential of polyesterase activity within different families of the order Enterobacterales.

The esterases identified in the genomes of Hal280-CE (*Photobacterium* sp.) and Hal099-E (*Pseudoalteromonas* sp.) are related to the verified PET–active enzymes Mors1 [75] and PmC [77], respectively. The association of the putative esterase from Hal280-CE to Mors1 presented low similarity (30%, results from the MSA), while having a conserved region for the pentapeptide motif G-x1-S-x2-G, where the catalytic active Ser is present. The amino acid sequence similarity between Hal099-E to PmC was of 19%. Like Hal280-CE, Hal099-E shows also the pentapeptide motif. The presence of the conserved G-x1-S-x2-G-motif and the close relationship to previously described PET–active enzymes provide evidence that Hal099-E and Hal280-CE are putative esterases potentially responsible for the BHET–activity in the associated strains.

## 5. Conclusions

This study conducted an in–depth screening of marine bacterial strains from the order Enterobacterales. The different isolation sources from the investigated strains supported an exploration of potential polyesterase activity from a variety of marine environments. The use of standardized screening methods has proven to be suitable for determining polyesterase activity. The results of this study indicate that bacteria isolated from sponges and bryozoans could be a promising source of polyesterase activity. Symbiotic and holobiont organisms may be valuable potential candidates for bioremediation of plastic pollution, which could facilitate a move towards a more sustainable blue marine economy model [4],[22]. The large number of orthologous proteins associated with α/β–superfamily hydrolases found in this study suggests that polyesterases may be more diverse than previously reported, and encompass a broad repertoire of active sites [80]. Furthermore, the added value of experimental assays supported by computational tools has revealed the potential of marine bacteria from the order Enterobacterales for PETase-like activity, indicating greater diversity and distribution of this particular type of enzymatic activity, as previously thought.
