# Supplementary material for: Bioprospecting for polyesterase activity relevant for PET degradation in marine Enterobacterales isolates
Source: AIMS Microbiol. 2023 Jun 15;9(3):518–39. doi: 10.3934/microbiol.2023027 (PMC10462454; doi:10.3934/microbiol.2023027)
Supplement: Supplementary file 1 [file microbiol-09-03-027-s001.pdf]

AIMS Microbiology

AIMS Microbiology, 9(3): 518–539.
DOI: 10.3934/microbiol.2023027
Received: 22 March 2023
Revised: 24 May 2023
Accepted: 07 June 2023
Published: 15 June 2023

http://www.aimspress.com/journal/microbiology

Research article

Bioprospecting for polyesterase activity relevant for PET degradation in marine Enterobacterales isolates

Denisse Galarza–Verkovitch[1], Onur Turak[1], Jutta Wiese[1], Tanja Rahn[1], Ute Hentschel[1,2] and Erik Borchert[1,*]

[1] GEOMAR Helmholtz Centre for Ocean Research Kiel, Kiel, Schleswig–Holstein, Germany
[2] Christian- Albrechts University of Kiel, Kiel, Schleswig–Holstein, Germany

* **Correspondence:** Email: mailto:eborchert@geomar.de; Pho: +494316004485; Fax: +494316004482

Supplementary Material

Table S1. Isolate screening results and associated data. Associated bacterial strains information and activity screening results. All isolates are grouped by family-level within the order Enterobacterales, and genus is specified. Screening results are included for all isolates tested on the lipid substrate, tributyrin (TRI), as well as the three polyester substrates, polycaprolactone-diol (PCD), polycaprolactone (PCL), and bis(2-Hydroxyethyl) terephthalate (BHET). In addition, source material and NCBI accession number of the 16S rRNA gene sequence is provided. Type-strain strain IDs are indicated by a superscript "T". Screening results are represented by symbolic coding, where: "-"= no activity; "+" = low activity; "++" = medium activity; "+++" = high activity. Marked with an asterisk (*) is a *Bacillus halotolerans* strain (DSM 8802^T) that was used as outgroup in Figure 2.

Strain ID	TRI	PCD	PCL	BHET	Family	Genus	Source	GenBank
B326	+	-	-	-	*Alteromonadaceae*	*Alteromonas*	Bryozoa	FN295790
B327	+	-	-	-	*Alteromonadaceae*	*Alteromonas*	Bryozoa	FN295791
B330	+	-	+	-	*Alteromonadaceae*	*Alteromonas*	Bryozoa	FN295794
B408	-	-	+	-	*Alteromonadaceae*	*Alteromonas*	Bryozoa	FN295809
M71_N19	-	-	-	-	*Alteromonadaceae*	*Alteromonas*	Water	OQ711989
M71_N85	++	-	-	-	*Alteromonadaceae*	*Alteromonas*	Water	OQ711994
M71_N51	+	-	-	-	*Alteromonadaceae*	*Alteromonas*	Water	OQ711993
M71_N41	+	-	-	-	*Alteromonadaceae*	*Alteromonas*	Water	OQ711992
M71_D33	++	-	-	-	*Alteromonadaceae*	*Alteromonas*	Sediment	FM99270
M71_D45	+	-	+	-	*Alteromonadaceae*	*Alteromonas*	Sediment	FM992719
M71_N36	+++	++	-	-	*Alteromonadaceae*	*Alteromonas*	Water	OQ711991
M71_D39	+	+	-	-	*Alteromonadaceae*	*Alteromonas*	Sediment	FM992717
M71_D56	+	+	-	-	*Alteromonadaceae*	*Alteromonas*	Sediment	FM992724
M71_N08	++	+	-	-	*Alteromonadaceae*	*Alteromonas*	Water	OQ711987
Y	++	++	++	-	*Alteromonadaceae*	*Alteromonas*	Biofilm	OQ712008
LD86	+	-	-	-	*Alteromonadaceae*	*Paraglaciecola*	Algae	AM913910

Continued on next page

Strain ID	TRI	PCD	PCL	BHET	Family	Genus	Source	GenBank
Hal342	++	+	++	+++	*Alteromonadaceae*	*Paraglaciecola*	Sponge	MT406721
Hal344	++	+	++	+++	*Alteromonadaceae*	*Paraglaciecola*	Sponge	MT406723
B129	++	+	++	+	*Alteromonadaceae*	*Pseudoalteromonas*	Bryozoa	FN295825
B130a	+	-	-	-	*Alteromonadaceae*	*Pseudoalteromonas*	Bryozoa	OQ711966
B131b	++	-	++	++	*Alteromonadaceae*	*Pseudoalteromonas*	Bryozoa	FN295827
B137	+	+	++	++	*Alteromonadaceae*	*Pseudoalteromonas*	Bryozoa	FN295828
B160	++	-	+++	+++	*Alteromonadaceae*	*Pseudoalteromonas*	Bryozoa	FN295747
B179	++	+	+++	++	*Alteromonadaceae*	*Pseudoalteromonas*	Bryozoa	FN295762
B193	+	-	+	++	*Alteromonadaceae*	*Pseudoalteromonas*	Bryozoa	FN295768
B281	+	-	+	-	*Alteromonadaceae*	*Pseudoalteromonas*	Bryozoa	FN295784
B28	++	+	++	++	*Alteromonadaceae*	*Pseudoalteromonas*	Bryozoa	FN295812
B62	++	-	++	++	*Alteromonadaceae*	*Pseudoalteromonas*	Bryozoa	FN295816
B22	+	-	-	-	*Alteromonadaceae*	*Pseudoalteromonas*	Bryozoa	FN295810
B35	+	+	-	-	*Alteromonadaceae*	*Pseudoalteromonas*	Bryozoa	FN295813
B49	+	+	+	-	*Alteromonadaceae*	*Pseudoalteromonas*	Bryozoa	FN295814
B169	-	-	-	-	*Alteromonadaceae*	*Pseudoalteromonas*	Bryozoa	FN295757
B182	+	-	-	-	*Alteromonadaceae*	*Pseudoalteromonas*	Algae	OQ711967
B201	++	-	-	-	*Alteromonadaceae*	*Pseudoalteromonas*	Bryozoa	FN295770
B207	++	+	++	-	*Alteromonadaceae*	*Pseudoalteromonas*	Hydrozoa	OQ711970
B214	+	-	+	-	*Alteromonadaceae*	*Pseudoalteromonas*	Hydrozoa	OQ711975
B296	+	-	-	-	*Alteromonadaceae*	*Pseudoalteromonas*	Bryozoa	FN295786
E Sponge3 (785)	++	-	-	-	*Alteromonadaceae*	*Pseudoalteromonas*	Sponge	OQ711980
Hal024	+	+	-	-	*Alteromonadaceae*	*Pseudoalteromonas*	Sponge	MT406405
Hal040	+	+	+++	+++	*Alteromonadaceae*	*Pseudoalteromonas*	Sponge	MT406421

Continued on next page

Strain ID	TRI	PCD	PCL	BHET	Family	Genus	Source	GenBank
Hal056	++	+	+++	+++	*Alteromonadaceae*	*Pseudoalteromonas*	Sponge	MT406437
Hal099	+++	+	+++	+++	*Alteromonadaceae*	*Pseudoalteromonas*	Sponge	MT406481
Hal242	+	-	+	-	*Alteromonadaceae*	*Pseudoalteromonas*	Sponge	MT406626
Hal273	++	++	+++	+++	*Alteromonadaceae*	*Pseudoalteromonas*	Sponge	MT406657
L28	+	-	-	-	*Alteromonadaceae*	*Pseudoalteromonas*	Algae	AM913889
L230	++	-	+	-	*Alteromonadaceae*	*Pseudoalteromonas*	Algae	AM913882
L232a	+	-	-	-	*Alteromonadaceae*	*Pseudoalteromonas*	Algae	AM913936
M71_D105	++	-	-	-	*Alteromonadaceae*	*Pseudoalteromonas*	Sediment	FM992739
M71_D114	+	-	-	-	*Alteromonadaceae*	*Pseudoalteromonas*	Sediment	FM992791
M71_D76	+	+	-	-	*Alteromonadaceae*	*Pseudoalteromonas*	Sediment	FM992730
M71_N133	+	-	-	-	*Alteromonadaceae*	*Pseudoalteromonas*	Water	OQ711988
M71_W03x	++	+	-	-	*Alteromonadaceae*	*Pseudoalteromonas*	Water	FM992785
M71_N28	+	-	-	-	*Alteromonadaceae*	*Pseudoalteromonas*	Water	OQ711990
LD85	++	-	-	-	*Enterobacteriaceae*	*Escherichia*	Algae	OQ711984
Hal018	+	+	+	-	*Enterobacteriaceae*	*Thalassomonas*	Sponge	MT406399
DSM 3368^{T}	+	++	++	+	*Morganellaceae*	*Photorhabdus*	Nematode	X82248
DSM 16336^{T}	-	-	-	-	*Morganellaceae*	*Xenorhabdus*	Nematode	AJ810292
DSM 16337^{T}	-	-	-	-	*Morganellaceae*	*Xenorhabdus*	Nematode	AJ810294
DSM 17910^{T}	++	+	-	-	*Morganellaceae*	*Xenorhabdus*	Nematode	DQ211717
DSM 3370^{T}	+	-	+	-	*Morganellaceae*	*Xenorhabdus*	Nematode	D78009
DSM 4766^{T}	-	-	-	-	*Morganellaceae*	*Xenorhabdus*	Nematode	X82252
B213	+	+	-	-	*Shewanellaceae*	*Shewanella*	Sponge	OQ711974

Continued on next page

Strain ID	TRI	PCD	PCL	BHET	Family	Genus	Source	GenBank
B147	+	+	+	-	*Shewanellaceae*	*Shewanella*	Bryozoa	FN295750
B153	++	-	-	-	*Shewanellaceae*	*Shewanella*	Bryozoa	FN295749
B157	+	-	+	-	*Shewanellaceae*	*Shewanella*	Bryozoa	FN295752
B159	+	+	-	-	*Shewanellaceae*	*Shewanella*	Bryozoa	FN295751
B165	++	-	-	-	*Shewanellaceae*	*Shewanella*	Bryozoa	FN295766
B171	+	+	-	-	*Shewanellaceae*	*Shewanella*	Bryozoa	FN295764
B186	+	+	-	-	*Shewanellaceae*	*Shewanella*	Algae	OQ711968
B191	++	-	-	-	*Shewanellaceae*	*Shewanella*	Algae	OQ711969
B194	++	-	-	-	*Shewanellaceae*	*Shewanella*	Bryozoa	FN295773
B209	+	-	-	-	*Shewanellaceae*	*Shewanella*	Hydrozoa	OQ711971
B210	+	-	-	-	*Shewanellaceae*	*Shewanella*	Hydrozoa	OQ711972
B211	+	-	-	-	*Shewanellaceae*	*Shewanella*	Hydrozoa	OQ711973
B219	+	-	+	-	*Shewanellaceae*	*Shewanella*	Hydrozoa	OQ711976
B221	++	-	-	-	*Shewanellaceae*	*Shewanella*	Hydrozoa	OQ711977
B224b	+	-	-	-	*Shewanellaceae*	*Shewanella*	Hydrozoa	OQ711978
B225	+	-	-	-	*Shewanellaceae*	*Shewanella*	Bryozoa	FN295775
B237a	+	-	+	-	*Shewanellaceae*	*Shewanella*	Bryozoa	FN295774
B246	+	-	-	-	*Shewanellaceae*	*Shewanella*	Bryozoa	FN295778
B256	++	-	-	-	*Shewanellaceae*	*Shewanella*	Hydrozoa	OQ711979
B163	+	-	-	-	*Shewanellaceae*	*Shewanella*	Bryozoa	FN295765
DSM 16474	+	-	+	-	*Shewanellaceae*	*Shewanella*	Nematode	OR016173
Hal001	+	+	-	-	*Shewanellaceae*	*Shewanella*	Sponge	MT406382
Hal006	-	-	-	-	*Shewanellaceae*	*Shewanella*	Sponge	MT406387
Hal027	+	+	+	-	*Shewanellaceae*	*Shewanella*	Sponge	MT406408
Hal250	+	+	-	-	*Shewanellaceae*	*Shewanella*	Sponge	MT406634

Continued on next page

Strain ID	TRI	PCD	PCL	BHET	Family	Genus	Source	GenBank
Hal360	++	+	+	-	*Shewanellaceae*	*Shewanella*	Sponge	OQ711981
Hal374	+	-	+	-	*Shewanellaceae*	*Shewanella*	Sponge	OQ711983
L171	+	-	+	-	*Shewanellaceae*	*Shewanella*	Algae	AM913976
LMG 23746^T	+	-	-	-	*Shewanellaceae*	*Shewanella*	Fish	AB681675
PP-He15 brown	++	+	+	+	*Shewanellaceae*	*Shewanella*	Fish	OQ711996
PP-He22b	+	-	+	-	*Shewanellaceae*	*Shewanella*	Fish	OQ711998
PP-Sp27a-2	++	-	++	+	*Shewanellaceae*	*Shewanella*	Fish	OQ711999
PP-Sp5	+	+	++	-	*Shewanellaceae*	*Shewanella*	Fish	OQ712000
PP-Sp8	+	+	-	-	*Shewanellaceae*	*Shewanella*	Fish	OQ712001
PP-X12	+	++	+	-	*Shewanellaceae*	*Shewanella*	Sediment	OQ712002
PP-Z4	++	+	-	-	*Shewanellaceae*	*Shewanella*	Sediment	OQ712006
Hal366	+	-	+	-	*Vibrionaceae*	*Photobacterium*	Sponge	OQ711982
Hal280	+	+	+	+	*Vibrionaceae*	*Photobacterium*	Sponge	MT40666
B105	+	-	-	-	*Vibrionaceae*	*Vibrio*	Bryozoa	FN295822
B142	++	-	-	-	*Vibrionaceae*	*Vibrio*	Bryozoa	FN295830
B96a	+	+	-	-	*Vibrionaceae*	*Vibrio*	Bryozoa	OQ711965
B80	++	+	+	-	*Vibrionaceae*	*Vibrio*	Bryozoa	FN295821
B109	-	-	-	-	*Vibrionaceae*	*Vibrio*	Bryozoa	FN295823
DSM 19607^T	++	++	++	+	*Vibrionaceae*	*Vibrio*	Cnidaria	MG722658
DSM 23640^T	+++	+	-	-	*Vibrionaceae*	*Vibrio*	Sponge	GU223601

Continued on next page

Strain ID	TRI	PCD	PCL	BHET	Family	Genus	Source	GenBank
DSM 24595^T	+	-	-	-	*Vibrionaceae*	*Vibrio*	Fish	JF316656
DSM 25438^T	++	+	-	-	*Vibrionaceae*	*Vibrio*	Algae	MT760286
DSM 507^T	+	-	-	-	*Vibrionaceae*	*Vibrio*	Biofilm	X70640
Hal025	++	+	+	-	*Vibrionaceae*	*Vibrio*	Sponge	MT406406
Hal054	+	-	+	+++	*Vibrionaceae*	*Vibrio*	Sponge	MT406435
Hal086	+	+	+	-	*Vibrionaceae*	*Enterovibrio*	Sponge	MT406467
Hal108	+	+	-	-	*Vibrionaceae*	*Enterovibrio*	Sponge	MT406490
Hal110	+	++	+	+	*Vibrionaceae*	*Enterovibrio*	Sponge	MT406492
Hal281	+	+	-	-	*Vibrionaceae*	*Vibrio*	Sponge	MT406664
LD156	++	+	-	-	*Vibrionaceae*	*Vibrio*	Algae	MT406665
M60_M31a	+	+	+	+	*Vibrionaceae*	*Vibrio*	Sediment	OQ711985
M60_M70	++	++	++	+	*Vibrionaceae*	*Vibrio*	Mussel	OQ711986
M71_N92a	++	+	++	+	*Vibrionaceae*	*Vibrio*	Water	OQ711995
PP-He21	++	+	+	-	*Vibrionaceae*	*Vibrio*	Fish	OQ711997
PP-XX3	-	-	-	-	*Vibrionaceae*	*Vibrio*	Sediment	OQ712003
PP-XX4-1	-	-	+	-	*Vibrionaceae*	*Vibrio*	Sediment	OQ712004
PP-XX7	-	+++	+	+	*Vibrionaceae*	*Vibrio*	Sediment	OQ712005
SA48	++	+	++	+	*Vibrionaceae*	*Vibrio*	Biofilm	OQ712007
DSM 8022^T*	+	+	++	-	*Bacillaceae*	*Bacillus*	Soil	MN483266

Table S2. MB-PETnp activity screening results. The strains that tested positive for MB-BHET were subsequently screened for PET activity. Strains exhibiting a reflection on the media are denoted with an asterisk (*).

Strain ID	Closest–relative according to GTDB-Tk	PETnp
B129*	*Pseudoalteromonas* sp.	+
B131b*	*Pseudoalteromonas* sp.	++
B137	*Pseudoalteromonas* sp.	-
B160	*Pseudoalteromonas* sp.	-
B179*	*Pseudoalteromonas* sp.	++
B28	*Pseudoalteromonas* sp.	+
B62*	*Pseudoalteromonas* sp.	+
B193	*Pseudoalteromonas* sp.	+++
DSM 3368^{T}	*Photorhabdus luminescens*	-
DSM 19607^{T}	*Vibrio coralliilyticus*	-
Hal110	*Enterovibrio sp.*	-
Hal040	*Pseudoalteromonas* sp.	+++
Hal054	*Vibrio* sp.	-
Hal056	*Pseudoalteromonas* sp.	+++
Hal099	*Pseudoalteromonas* sp.	+++
Hal273	*Pseudoalteromonas* sp.	+++
Hal280	*Photobacterium* sp.	-
Hal342*	*Paraglaciecola* sp.	+
M60_M31a	*Vibrio* sp.	-
M60_M70	*Vibrio* sp.	-
PP-He15brown	*Shewanella* sp.	-
PP-Sp27a-2	*Shewanella* sp.	-
PP-XX7	*Vibrio* sp.	-
SA48	*Vibrio* sp.	-

Table S3. Weight change tested on PET foils from significant strains. Listed is the weight change after 30 days incubation at room temperature (21°C).

Strain ID	Closest–relative according to GTDB-Tk	Weight [mg] T_0	Weight [mg] T_1
B129	*Pseudoalteromonas* sp.	29,2	29,5
B131b	*Pseudoalteromonas* sp.	29,1	29,7
B137	*Pseudoalteromonas* sp.	30,8	31,1
B179	*Pseudoalteromonas* sp.	32	32,3
B28	*Pseudoalteromonas* sp.	31,3	31,5
B62	*Pseudoalteromonas* sp.	30,1	30,2
B193	*Pseudoalteromonas* sp.	30,3	30,6
Hal040	*Pseudoalteromonas* sp.	28,7	28,8
Hal056	*Pseudoalteromonas* sp.	31,3	31,6
Hal099	*Pseudoalteromonas* sp.	32,3	32,6
Hal273	*Pseudoalteromonas* sp.	29,4	29,7
Hal280	*Photobacterium* sp.	32,8	33,1
Hal342	*Paraglaciecola* sp.	29,3	30
		Controls	
	Only MB+PET	34,6	36,6

Table S4. Genome assembly statistics. Values revealing the genome purity and completeness from the 23 genomes analyzed, along with values of its size, G-C content, N50, and predicted genes are shown. Strains marked in bold and marked with *showed N50 lower values < 1Mb.

Strain ID	Completeness (%)	Contamination (%)	G-C content (%)	N50 (Kb)	Genome size (Mb)	No. of contigs	Predicted genes
B129	99.92	0.17	39.34	3798533	4 619 747	2	4 217
B131b	99.19	0.22	38.97	3928489	4 799 075	2	4 596
B137	97.80	0.28	39.23	3805162	4 664 055	3	4 841
B160	99.11	0.23	40.41	3995703	4 990 457	2	4 809
B28	97.78	0.24	39.26	3813810	4 624 011	2	5 132
B62	99.20	0.47	39.10	4067232	5 053 000	7	4 879
B193	77.86	0.99	39.46	2881883	4 624 656	9	7800
DSM 19607^T	99.28	0.60	45.71	3452511	5 719 173	3	5 307
DSM 3368^T*	99.46	0.54	42.93	530 955	5 464 970	16	4670
Hal110*	92.69	0.00	47.80	141940	5 553 615	72	5 575
Hal040	99.67	0.25	41.45	3788806	4 665 450	2	4 282
Hal054	99.36	2.37	43.6	3417947	5 026 408	3	4 674
Hal056	99.23	0.25	41.46	3788601	4 665 210	2	4330
Hal099*	73.01	0.81	41.08	84834	4 093 015	75	7 334
Hal273	99.86	0.88	39.38	3767911	4 655 783	3	4 287
Hal280	97.23	0.45	51.07	2902238	4 798 543	6	4 548

Continued on next page

Strain ID	Completeness (%)	Contamination (%)	G-C content (%)	N50 (Kb)	Genome size (Mb)	No. of contigs	Predicted genes
Hal342	92.79	1.91	44.10	1512141	5 568 427	16	7 365
M60_M31a	72.66	1.68	45.04	113224	4 551 125	61	6 834
M60_M70*	99.68	0.54	44.77	3371361	5 287 758	3	4 887
PP_He15brown	99.73	0.25	46.21	5282401	5 350 725	3	4 819
PP-Sp27a-2	96.29	0.12	46.12	5118085	5 456 064	4	5 651
PP-XX7*	62.37	1.25	44.87	79420	4 656 231	95	7 987
SA48	97.64	0.75	42.82	3314225	4 412 949	20	4 405

Table S5. Reference dataset of functionally verified polyesterases activity on PET or PET model substrates from Proteobacteria. Sequences were obtained from the PAZy database [68], which were used to conduct BLASTP protein homology searches against the annotated proteins of interest and for further phylogenetic inferences.

Enzyme	Source	EC number	NCBI accession	References
ISF6_4831	*Ideonella sakaiensis* strain201-F6	EC 3.1.1.101	WP_054022242.1	[28]
PET12	*Polyangium brachysporum*	EC 3.1.1.1	WP_047194864.1	[26]
LCC	Uncultured bacterium (metagenome)	EC 3.1.1.74	AEV21261.1	[31]
PET5	*Oleispira antarctica* RB-8	EC 3.1.1.1	CCK74972.1	[26]
PET6	*Vibrio gazogenes* ATCC 43942	EC 3.1.1.1	WP_021018894.1	[26]
Mors1	*Moraxella* sp. TA144	EC 3.1.1.3	P19833.1	[75]
PE-H	*Pseudomonas aestusnigri*	EC 3.1.1.1	WP_105645204.1	[76]
PmC	*Pseudomonas mendocina*	EC 3.1.1.3	2FX5_A	[77]
MG8	*Pseudomonas* sp.	EC 3.1.1.1	MGYP000532440779	[78]
PpEst	*Pseudomonas pseudoalcaligenes*	EC 3.1.1.2	ANP21911.1	[79]

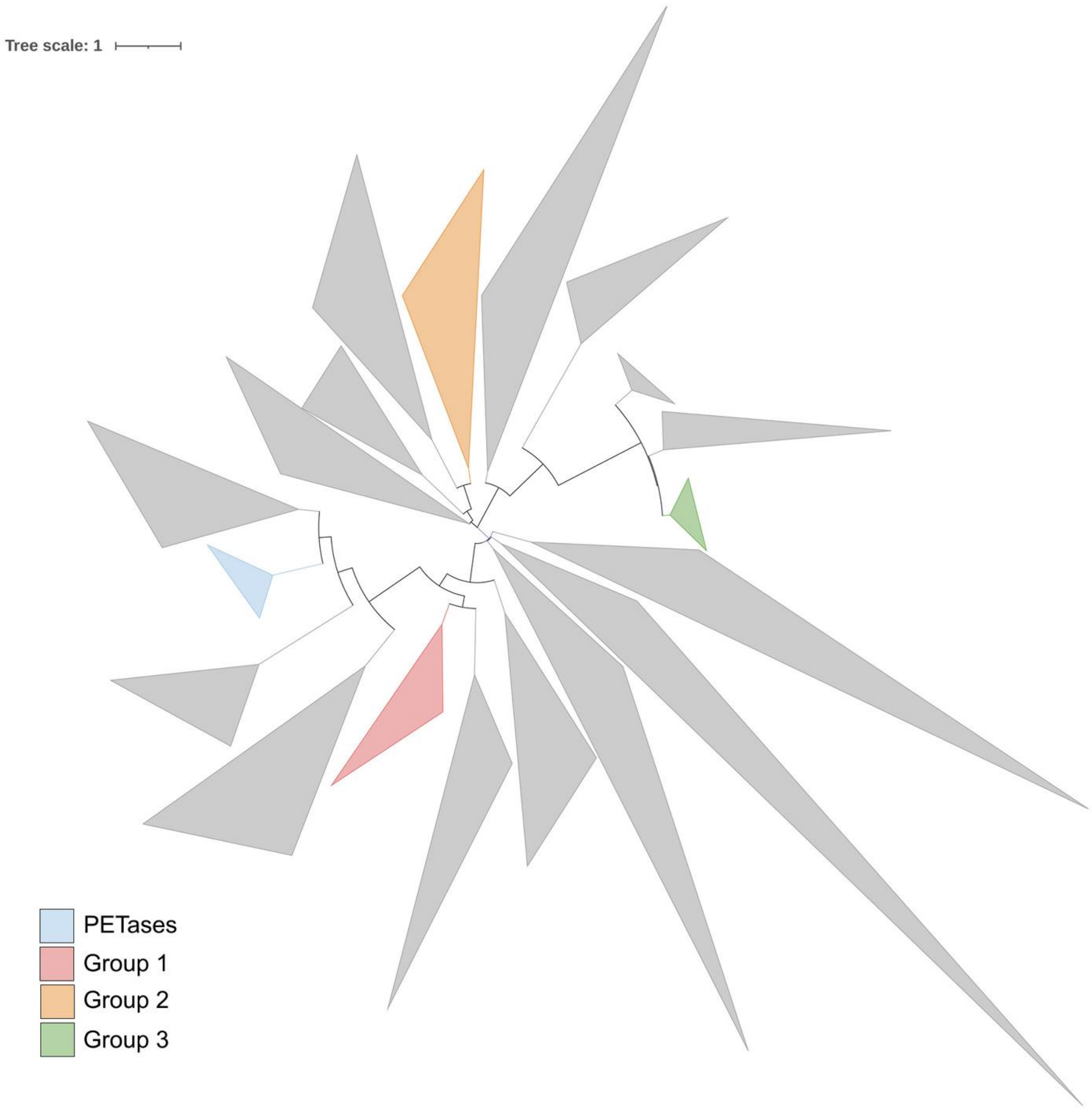

Figure S6. Protein ortholog sequence tree of α/β hydrolases superfamily affiliated sequences and PETase references obtained from the Egg-NOG mapper. Maximum likelihood (ML) phylogenetic tree calculated with 1000 bootstrap replicates, under best-fit substitution model with WAG + G model using MEGA-X and displayed with the iTOL online tool. Four groups of proteins are highlighted, with the reference PETases clustered together in light blue. Group 1 is associated with *Is*PETase [28], while group 2 is with PmC [77]. Group 3 is linked to PHB depolymerase activity. Detailed information on the protein sequences is listed in Table 3.

Table S7. SignalP-5.0 results for Hal099-E and H280-CE. Predicted signal peptides were utilized to confirm extracellular activity. They are classified into four categories. The prediction hit produced by SignalP is highlighted in bold.

Protein sequence	Protein type			
	Signal peptide (Sec/SPI)	TAT signal peptide (Tat/SPI)	Lipoprotein signal peptide (Sec/SPII)	Other
Hal099-E	0.0005	0.0001	**0.9992**	0.0002
Hal280-CE	0.0134	0.0367	0.0043	**0.9456**

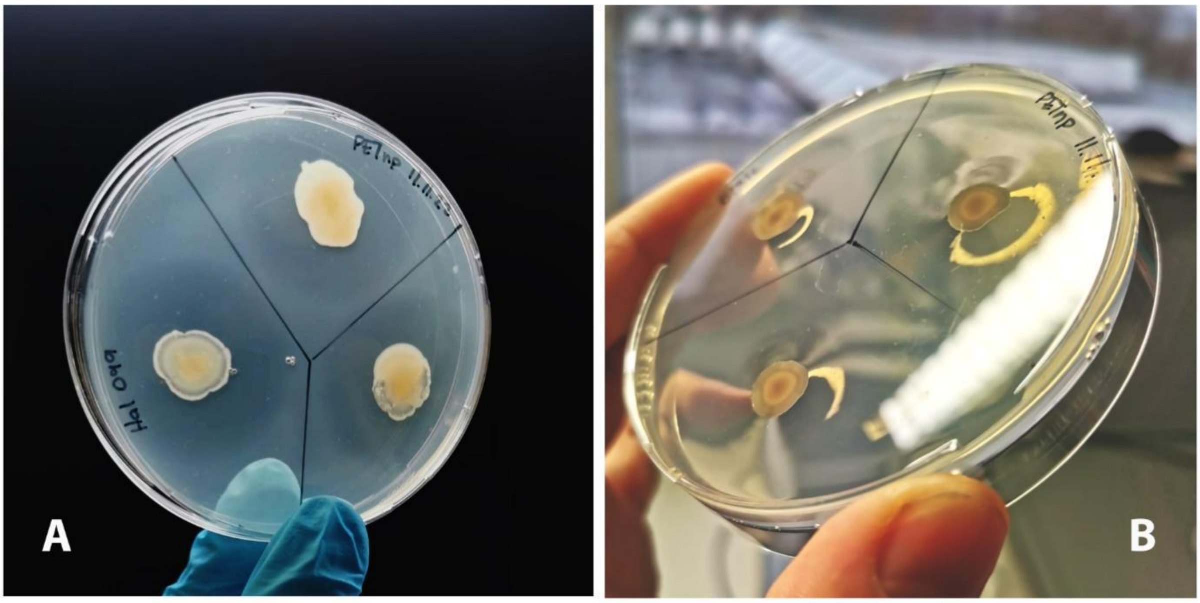

Figure S8. Results from MB-PETnp activity screening. Figure A illustrates Hal099 strain exhibiting a notable halo formation, while Figure B depicts a reflection encompassing the activity halo. This observation suggests a potential interaction between the PETnp and MB media, which may not be directly associated with polyesterase activity.

AIMS Press

© 2023 the Author(s), licensee AIMS Press. This is an open access article distributed under the terms of the Creative Commons Attribution License (http://creativecommons.org/licenses/by/4.0)
